# Maternal Syphilis: An Independent Risk Factor for Mother to Infant Human Immunodeficiency Virus Transmission

**DOI:** 10.1097/OLQ.0000000000000622

**Published:** 2017-04-17

**Authors:** Aarti Kinikar, Nikhil Gupte, Jayalakshmi Bhat, Renu Bharadwaj, Vandana Kulkarni, Ramesh Bhosale, Katherine N McIntire, Vidya Mave, Nishi Suryavanshi, Sandesh Patil, Robert Bollinger, Amita Gupta

**Affiliations:** From the *Byramjee Jeejeebhoy Government Medical College and Sassoon General Hospital, Pune, Maharashtra, India; †Division of Infectious Diseases, Johns Hopkins University School of Medicine, Baltimore, MD; ‡Byramjee Jeejeebhoy Government Medical College-Johns Hopkins University Clinical Research Site, Pune, Maharashtra, India; and §International Health, Johns Hopkins Bloomberg School of Public Health, Baltimore, MD

## Abstract

Syphilis is associated with increased human immunodeficiency virus acquisition and sexual transmission; we examined impact on human immunodeficiency virus mother-to-child transmission among mother-infant pairs enrolled in the India Six-Week Extended-Dose Nevirapine study. Maternal syphilis, diagnosed serologically using Venereal Disease Research Laboratory titer plus Treponema Pallidum Hemagglutination Assay, was associated with 2.5-fold greater risk.

## Abstract 2

A study of human immunodeficiency virus–infected pregnant women in India identified maternal syphilis co-infection as an independent risk factor for human immunodeficiency virus mother-to-child transmission by age 6 months with nearly 2.5-fold increased risk.

Although curable with antibiotics, syphilis remains a public health challenge, particularly for exposed infants. Worldwide, the World Health Organization (WHO) estimates 36.4 million syphilis cases among adults of reproductive age (15–49 years); women account for 47% of the estimated 12 million new cases each year, including two million cases during pregnancy.^1^ Maternal syphilis prevalence varies from 0.09% to 8.4% in different regions,^2^ yet surveillance estimates fail to capture the severity largely due to underreporting and suboptimal treatment, particularly in developing countries.^3,4^

In 2012, an estimated 950,000 maternal syphilis infections resulted in 360,000 adverse outcomes.^3^ Prior WHO reports associate up to 80% of cases with poor infant outcomes, including mortality, neonatal infections, and low birth weight.^2^ In high human immunodeficiency virus (HIV)/syphilis burden regions, such as Africa and Southeast Asia, syphilis may also place exposed pregnancies at increased risk of HIV mother-to-child transmission (MTCT).

Syphilis pathogenesis breaches the genital wall epithelium and may facilitate HIV virus entry into the bloodstream. Although studies have associated syphilis with increased HIV acquisition^5^ and sexual transmission,^6^ previous studies of perinatal HIV transmission have been limited by a variety of factors and have yielded mixed results.^6–11^ Identifying maternal syphilis as an independent risk factor for HIV MTCT would suggest an important, modifiable target to be prioritized and addressed by prevention of MTCT (PMTCT) programs.

Currently, up to one third of antenatal care (ANC) attendees are not tested for syphilis,^3,4^ and many cases remain untreated or improperly treated. Access to treatment remains limited in many developing countries^2,4^ and in some high burden countries, including India where only 70% of women receive ANC at different levels of the health system.^12^ Evidence linking maternal syphilis and HIV MTCT could supplement existing outcome and surveillance data to secure increased financial and political support for integrated strategies for elimination of syphilis and HIV MTCT, including new point-of-care diagnostics.

## MATERIALS AND METHODS

We conducted a secondary analysis of HIV-infected pregnant women and their infants who were enrolled in the India Six-Week Extended-Dose Nevirapine (SWEN) study, a National Institutes of Health (NIH)-funded phase III randomized controlled trial of extended infant nevirapine prophylaxis for prevention of breast milk HIV MTCT. The primary outcome was HIV-1 transmission at age 6 months; key secondary outcomes were to assess risk factors for HIV MTCT, including maternal co-infections, such as syphilis. Institutional review boards from Johns Hopkins University and the Byramjee Jeejeebhoy Government Medical College Ethics Committee approved the SWEN study methods, which are described elsewhere.^13^

Maternal syphilis was defined serologically; all mothers underwent screening at the first or second study visit using routine venereal disease research laboratory (VDRL) titer followed by confirmatory *Treponema pallidum* hemagglutination assay (TPHA). Mothers with reactive VDRL and TPHA assay tests were treated immediately with penicillin as per the national program. HIV-1 transmission was assessed at 48 hours post delivery and at each study visit (except weeks 3 and 5) using an in-house, externally validated HIV DNA polymerase chain reaction (PCR) assay developed at the National acquired immune deficiency syndrome (AIDS) Research Institute in Pune, India; a positive test was confirmed by HIV-1 RNA PCR at the next study visit. Due to concerns that prophylactic nevirapine might partially suppress viral load, 5000 copies/mL was used to define a positive HIV RNA PCR study (instead of 10,000 copies/mL) based on a modification of the Pediatric AIDS Clinical Trials Group definition for infant HIV diagnosis.^13^

Data were analyzed using STATA (version 12.0). Continuous variables are summarized using median and interquartile range (IQR) and compared using the Mann–Whitney rank-sum test. Maternal CD4 cell count and breastfeeding duration are categorized into clinically meaningful groups and compared using Fisher exact test. *P* values of 0.05 or less were deemed statistically significant. Univariable and multivariable Cox-proportional hazards models were used to assess the independent effect of maternal syphilis on time to HIV MTCT; follow-up was truncated at 6 months. Survival curves by syphilis coinfection status are nonintersecting hence satisfy Cox-regression assumptions. The analysis was adjusted for potential confounders that were statistically significant in univariate analysis as well as factors known to impact HIV transmission.

## RESULTS

A total of 658 HIV-infected mothers underwent syphilis screening and delivered live-born infants (Table 1). Median maternal CD4 count and HIV RNA load closest to delivery were 454 cells/mm^3^ (IQR, 312–648) and 3.73 log_10_ copies/mL (IQR, 2.91–4.52), respectively. A majority of women received ANC for a median of 8 weeks before delivery (IQR, 5–12), underwent vaginal delivery and received intrapartum nevirapine; a minority received antepartum zidovudine or highly active antiretroviral therapy (HAART) during follow-up, and 29 (4%) mothers had tuberculosis coinfection. Median birth weight was 2650 g (IQR, 2450–3000), 326 (50%) infants received SWEN, and median breastfeeding duration was 104 days (IQR, 98–183 days).

**TABLE 1:**
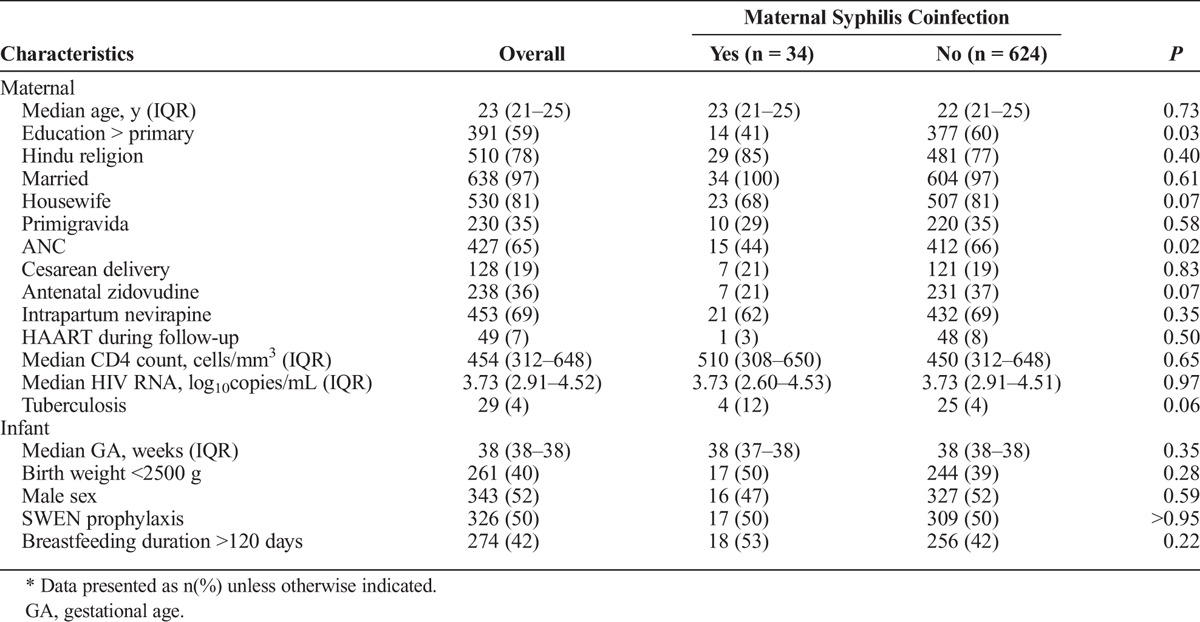
Maternal and Infant Characteristics in Overall Cohort (n = 658) and by Maternal Syphilis Coinfection Status*

Of 34 (5%) syphilis coinfected mothers, 100% received penicillin. Syphilis diagnosis occurred at a median of 29 days before delivery (IQR, −59 to 3) with VDRL titers from 1:1 to 1:64; 18 (53%) mothers had titers ≥ 1:16, and 16 had titers <1:8 (data not shown). Mother-infant pairs were largely similar by maternal syphilis coinfection status (Table 1). Syphilis coinfected mothers were more likely to have a lower education level (*P* = 0.03) and less likely to have received ANC (*P* = 0.02).

**Figure 1:**
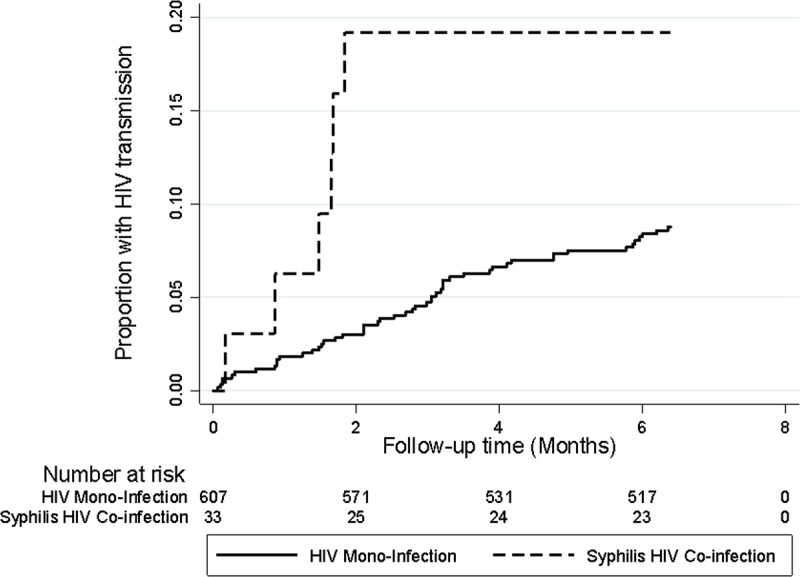
Kaplan-Meier estimated risk of HIV-1 transmission at age 6 months by maternal syphilis HIV coinfection status. The Kaplan-Meier plot shows a higher cumulative probability of HIV-1 transmission by 6 months in maternal syphilis HIV co-infection (dashed line) compared to maternal HIV monoinfection (solid line). Infant HIV-1 infection was assessed at weeks 1, 2, 4, 6, 10, 14, and 6 months.

A total of 67 (10%) infants were HIV-1 infected by 6 months. Maternal-infant characteristics by HIV transmission status have been previously described.^13^ HIV-1 transmission occurred in 7 (21%) of 34 syphilis-exposed infants compared to 60 (10%) of 624 unexposed infants; of these, 5 (15%) syphilis exposed infants and 21 (3%) unexposed infants were HIV-infected at birth. Kaplan-Meier estimated cumulative probability of HIV-1 transmission was higher in maternal syphilis HIV coinfection than HIV monoinfection (19.2%; 95% confidence interval [CI], 9.1%–37.8% vs 8.8%; 95% CI, 6.7%–11.4%) (Fig. 1). In multivariate analysis that adjusted for known maternal and infant risk and protective factors associated with HIV MTCT, maternal syphilis HIV coinfection was associated with 2.43-fold (95% CI, 1.004–5.89; *P* = 0.049) and 2.53-fold (95% CI, 1.05–6.10; *P* = 0.04) increased hazards of HIV-1 transmission in models that included and excluded maternal HAART antecedent to HIV transmission, respectively (Table 2).

**TABLE 2:**
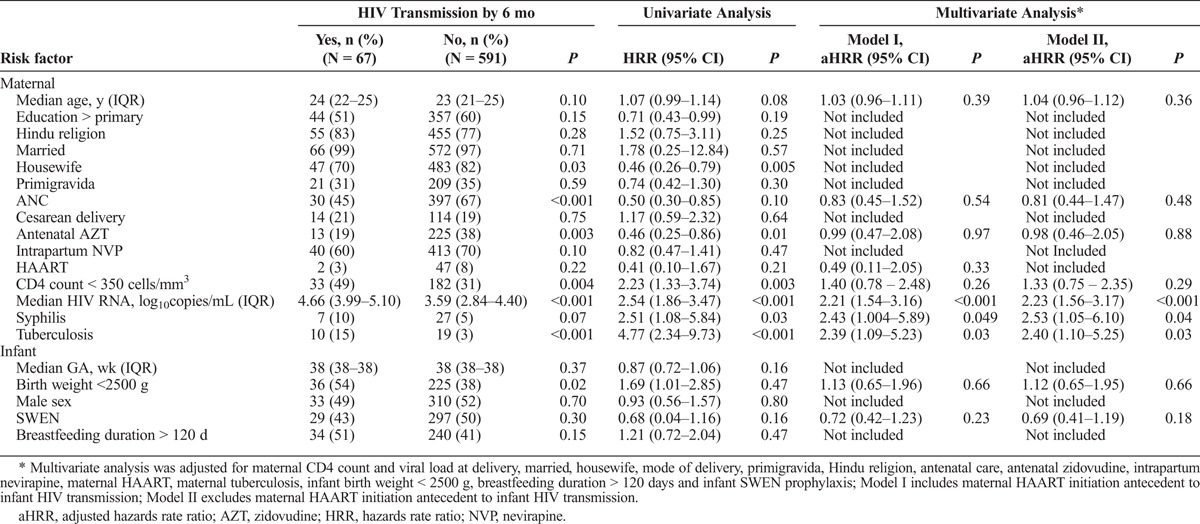
Results of Cox Proportional Analysis to Identify Risk Factors Associated With Mother-to-Child HIV-1 Transmission in a Cohort of HIV-Infected Pregnant Women Screened for Maternal Syphilis in Pune, India (n = 658)

## DISCUSSION

Our secondary analysis of mother-infant pairs enrolled in the India SWEN study identifies maternal syphilis as an independent risk factor for HIV MTCT with approximately 2.5-fold increased risk. Maternal syphilis is potentially preventable and treatable using various cost-effective methods, including health education, screening, and early diagnosis and treatment of pregnant women and their partners.^14,15^ Our findings provide new evidence to support the prioritization of maternal syphilis screening, particularly via integrated syphilis/HIV services in ANC settings.

Data assessing syphilis as a risk factor for HIV MTCT are limited. Although generally consistent with previous studies associating syphilis with 2- to 9-fold and 2- to- 4-fold increased HIV sexual transmission and acquisition, respectively,^16^ our observed association between maternal syphilis and HIV MTCT is consistent with only 3^6–8^ of 6 prior studies of perinatal transmission.^6–11^ Notably, nondifferential misclassification bias may explain the negative findings of two conflicting studies, as genital ulcers^9^ or rapid plasma reagin without a confirmatory test^10^ were used to define maternal syphilis; the third conflicting study assessed syphilis alongside Herpes simplex virus type 2 testing, not as a primary focus.^11^

Our analysis of the timing of neonatal infection implies perinatal transmission among syphilis-exposed infants. Possible explanations for the association of maternal syphilis with HIV MTCT in a breastfeeding population^16–18^ include syphilis-mediated breaches of maternal-fetal barriers and increased maternal infectiousness. In addition to breaches in genital wall epithelium, common histopathological abnormalities have been reported among placental and umbilical cord samples from infants of syphilis HIV coinfected women.^17^ *Treponema pallidum* or the host immune response to the bacteria may compromise the structural or functional integrity of the maternal-fetal barrier and facilitate vertical HIV transmission.

Immune activation and inflammation may also increase maternal infectiousness. This mechanism has been suggested to explain the associations of STIs and placental malaria with increased risk of HIV MTCT.^19^ Syphilis-mediated immune activation could increase HIV virus entry into the breast milk compartment, or as with many other infections, could result in increased maternal HIV viral load and decreased maternal CD4 count.^18,20^ We did not assess inflammation, but our group recently reported increased odds of HIV MTCT with increasing maternal soluble CD14 concentration in an overlapping cohort.^21^ Thus, there are several biologically plausible reasons for increased risk of HIV MTCT due to maternal syphilis and needs further exploration.

Our study has limitations. All factors that impact HIV MTCT may not have been accounted for; we did not measure placental malaria (prevalence was low in our population), chorioamnionitis,^22^ other STIs,^23^ or less commonly evaluated factors, such as maternal-infant human leukocyte antigen status or presence of CCR5D32 mutation. In addition, we did not examine the effect of syphilis stage or nontreponemal titer. None of the syphilis co-infected women in our cohort had lesions on physical exam (data not shown); diagnosis was based entirely on seroreactivity during routine antenatal screening. Early stage disease tends to be associated with greater acute inflammation and may predispose women to increased risk of vertical transmission.

Increased risk of HIV MTCT with maternal syphilis co-infection emphasizes the need to expand syphilis screening by integrating HIV and syphilis ANC services.^15,24^ Syphilis screening and treatment is a standard recommendation and is likely to be cost-effective by WHO-defined thresholds in a wide range of settings; current costs range from four to nineteen US dollars per disability-adjusted life year averted^25^ and would decrease if the effect of syphilis on HIV MTCT were considered. Implementation, however, has been patchy, which may be partially explained by the increased resources required to implement both universal HIV and syphilis testing in resource poor, high burden countries. Notably, financial resources are growing for HIV PMTCT through several donors, such as the Global Fund to fight AIDS, Tuberculosis and Malaria, and will enable scale-up of interventions, including antenatal HIV screening, particularly benefiting high prevalence countries with low service coverage and high health care cost.^18^ Further, a rapid dual immunoassay for syphilis and HIV recently yielded 100% dual specificity and sensitivity of 93.8% for HIV and 81.0% for syphilis in a NIH-funded field test.^26^ Investment in such point-of-care diagnostics may yield improved outcomes beyond syphilis in resource-poor countries.

Our analysis expands the high burden of maternal syphilis to include increased risk of HIV MTCT and is among the first to assess the relationship between maternal syphilis and HIV transmission in India, a country with the third highest global HIV burden. In addition to providing important data for Indian policy makers to ensure adequate resources for both syphilis and HIV testing among pregnant women, there is a broader need to: ensure access to ANC services; implement strategies for early syphilis detection and treatment at health care facilities; and identify and educate populations that do not access such facilities. Incorporating syphilis management into PMTCT programs would add little cost and have a major impact on child survival^14,15,24^; continued efforts to develop and disseminate a rapid, easily interpreted dual HIV/syphilis point-of-care assay may improve outcomes.
